# Risk factors for tuberculosis treatment failure, default, or relapse and outcomes of retreatment in Morocco

**DOI:** 10.1186/1471-2458-11-140

**Published:** 2011-02-28

**Authors:** Kelly E Dooley, Ouafae Lahlou, Iraqi Ghali, Janine Knudsen, My Driss Elmessaoudi, Imad Cherkaoui, Rajae El Aouad

**Affiliations:** 1 Johns Hopkins University School of Medicine, Baltimore, USA; 2 National Institute of Hygiene, Ministry of Health, Rabat, Morocco; 3 Moulay Youssef University Hospital, CHU Ibn Sina, Rabat, Morocco; 4 Johns Hopkins University, Baltimore, USA; 5 Pasteur Institute, Casablanca, Morocco

## Abstract

**Background:**

Patients with tuberculosis require retreatment if they fail or default from initial treatment or if they relapse following initial treatment success. Outcomes among patients receiving a standard World Health Organization Category II retreatment regimen are suboptimal, resulting in increased risk of morbidity, drug resistance, and transmission.. In this study, we evaluated the risk factors for initial treatment failure, default, or early relapse leading to the need for tuberculosis retreatment in Morocco. We also assessed retreatment outcomes and drug susceptibility testing use for retreatment patients in urban centers in Morocco, where tuberculosis incidence is stubbornly high.

**Methods:**

Patients with smear- or culture-positive pulmonary tuberculosis presenting for retreatment were identified using clinic registries in nine urban public clinics in Morocco. Demographic and outcomes data were collected from clinical charts and reference laboratories. To identify factors that had put these individuals at risk for failure, default, or early relapse in the first place, initial treatment records were also abstracted (if retreatment began within two years of initial treatment), and patient characteristics were compared with controls who successfully completed initial treatment without early relapse.

**Results:**

291 patients presenting for retreatment were included; 93% received a standard Category II regimen. Retreatment was successful in 74% of relapse patients, 48% of failure patients, and 41% of default patients. 25% of retreatment patients defaulted, higher than previous estimates. Retreatment failure was most common among patients who had failed initial treatment (24%), and default from retreatment was most frequent among patients with initial treatment default (57%). Drug susceptibility testing was performed in only 10% of retreatment patients. Independent risk factors for failure, default, or early relapse after initial treatment included male gender (aOR = 2.29, 95% CI 1.10-4.77), positive sputum smear after 3 months of treatment (OR 7.14, 95% CI 4.04-13.2), and hospitalization (OR 2.09, 95% CI 1.01-4.34). Higher weight at treatment initiation was protective. Male sex, substance use, missed doses, and hospitalization appeared to be risk factors for default, but subgroup analyses were limited by small numbers.

**Conclusions:**

Outcomes of retreatment with a Category II regimen are suboptimal and vary by subgroup. Default among patients receiving tuberculosis retreatment is unacceptably high in urban areas in Morocco, and patients who fail initial tuberculosis treatment are at especially high risk of retreatment failure. Strategies to address risk factors for initial treatment default and to identify patients at risk for failure (including expanded use of drug susceptibility testing) are important given suboptimal retreatment outcomes in these groups.

## Background

Tuberculosis (TB) continues to be a global public health problem, with an estimated 9.4 million incident cases of TB and 1.8 million deaths in 2008[1]. Drug resistance and obstacles to successful directly observed therapy short-course (DOTS) impede disease control. Among patients being retreated for TB because of initial treatment failure, default from initial treatment, or relapse following initial treatment, drug resistance is common and retreatment outcomes inferior[2,3].

Patients who fail, default from, or relapse after completion of standard first-line TB treatment and present for retreatment were previously grouped together by the World Health Organization (WHO) as Category II cases, and, in settings where individual drug susceptibility testing (DST) was not universally accessible, these patients were often treated with a standard retreatment regimen of first-line agents (a regimen that adds a single drug to the standard initial TB treatment regimen)[4]. Retreatment outcomes, however, are often poor, especially in patients with treatment failure or default[5]. DST may help identify those patients with multidrug-resistant (MDR) TB so that the appropriate antibiotics can be administered. Identifying local patient characteristics that confer higher risk of relapse, failure, or default from primary TB treatment may help inform country-specific prevention strategies aiming to reduce the need for retreatment, resulting in cost savings and diminished morbidity and transmission.

In Morocco, the incidence of TB in 2008 was 81 per 100,000 overall but was significantly higher in several urban centers, or "hot spots". Of the roughly 28,000 new TB cases nationally each year, 12% are retreatment cases[5]. National TB treatment guidelines in 2007 and 2008 recommended a Category I treatment regimen -- 2 months of isoniazid, rifampin, pyrazinamide, and streptomycin followed by 4 months of rifampin and isoniazid (2SHRZ/4RH) -- for new smear-positive cases and a Category II regimen -- 2HRZES/1RHEZ/5RHE (E = ethambutol) -- for retreatment cases. Beginning in 2009, ethambutol replaced streptomycin in Category I regimens. Among retreatment cases in Morocco, 12.2% are infected with *Mycobacterium tuberculosis *strains that are resistant to rifampin and isoniazid, or MDR TB[6]. National guidelines suggest drug susceptibility testing (DST) for all retreatment patients.

This retrospective cohort study examines the efficacy of standard TB retreatment in urban centers in Morocco, evaluates the uptake of DST testing for retreatment patients, and, using a nested case-control design, explores the risk factors that led to the need for retreatment in the first place.

## Methods

### Sites

Morocco's National Tuberculosis Program (NTP) is well-established. TB care is provided free of charge by the Ministry of Health. Regional TB programs are divided into sectors, and each sector has a public health center (CDTMR) staffed by a specialist in TB care. TB clinical care is provided at CDTMR, while TB medications are delivered via DOTS at local clinics or dispensaries. *M. tuberculosis *culture and DST are performed at the National Reference Laboratory at the National Institute of Hygiene (INH) in Rabat or at the regional reference laboratory at the Institute Pasteur in Casablanca (IPM). National guidelines recommend DST for all retreatment patients. We chose nine urban CDTMR in high-incidence settings in Rabat, Casablanca, Fes, Tangier, and El Jedida as sites.

### Design

A retrospective cohort study of retreatment cases focusing on patient characteristics, treatment outcomes, and drug susceptibility was conducted. To determine the risk factors for failure, relapse, or default after initial treatment that led to the need for retreatment, a nested case-control study was performed.

### Study population

Moroccan patients with smear- or culture-confirmed pulmonary TB presenting for retreatment between June 2007 and August 2008 were identified using clinic registries. Those who had failed initial treatment, had relapse after completing initial treatment, or defaulted after at least two months of initial treatment were included in the cohort study. Among patients in the cohort study, those whose initial treatment had ended within two years of starting their retreatment regimen were eligible to be included in the nested case-control study as cases. The rationale for the two-year limit was to make it more likely that initial treatment charts would be available, to ensure that standards of practice for initial TB treatment would be similar across cases, and to increase the likelihood that recurrent disease represented relapse rather than reinfection. Controls were chosen from among patients with successful initial treatment without failure, default, or early relapse selected from the same center and treatment week.

### Definitions

As per national guidelines, a patient with positive sputum smears for acid-fast bacilli after 5 months of continuous Category I treatment had treatment failure. A patient with initial treatment success after TB therapy of sufficient length (9 months for severe disease, 6 months for all others) that developed recurrent TB had treatment relapse. Treatment default was defined as interruption of treatment for ≥2 consecutive months. Treatment success was defined as treatment completion or cure. Retreatment patients were those receiving their first retreatment regimen after relapse, failure, or default.

### Data collection

DST results were reviewed at INH and IPM to identify sites that used DST testing services. At clinics chosen as study sites, the TB registry and medical records of included patients were reviewed. For retreatment patients, information from initial TB treatment was collected if initial treatment was within two years of retreatment, and this information was used for risk factor analyses. This study was approved by the Ministry of Health of Morocco and by the Institutional Review Board of the Johns Hopkins University School of Medicine.

### Drug Susceptibility Testing

Smear microscopy and culture were performed using standard methods. Specimens demonstrating growth of *M. tuberculosis *on Lowenstein-Jensen medium were tested for susceptibility to isoniazid, rifampin, ethambutol, and streptomycin using the proportion method. Critical concentrations were as follows: RIF 40 mcg/mL, INH 0.2 mcg/mL, streptomycin 4 mcg/mL, and ethambutol 2 mcg/mL. DST quality control was provided by the Supranational Laboratory at the Institute Pasteur of Algiers, as per standard laboratory operating procedures.

### Statistical analysis

Data analyses were performed in EpiInfo™ (Version 3.3.2, Centers for Disease Control, Atlanta, GA) except for multivariate logistic regressions, which were performed using STATA software, version 10.0 (StataCorp LP, College Station, Texas). Demographic and clinical characteristics of cases and controls during initial TB treatment were compared using Pearson's χ^2 ^or Fisher's exact tests for categorical variables and student's *t *tests for continuous variables. Variables known to be risk factors for relapse, failure, or default as well as factors found to be associated with these outcomes in univariate analyses were included in multivariable logistic regression models. Significance tests were two-sided with p-values of ≤ 0.05 considered statistically significant.

## Results

### Tuberculosis retreatment patients: population description, treatment outcomes, and DST results

291 patients with smear- or culture-positive pulmonary TB presenting for retreatment were identified and included in the study. Of retreatment cases, 232 (80%) had relapsed after completing an initial treatment regimen, 21 (7%) had failed an initial treatment regimen, and 38 (13%) had defaulted from initial treatment. The mean age was 37 years (IQR 27-46), and 78% were men. HIV and diabetes mellitus were rare (recorded for 1 and 3 patients, respectively).

A standard Category II retreatment regimen was used in 272 (93%) of 291 patients. Retreatment outcomes were as follows: 172 (59%) cured, 26 (9%) completed treatment, 7 (2%) died, 73 (25%) defaulted, and 11 (4%) failed. Retreatment was successful in 173 (74%) of relapse patients, 10 (48%) of failure patients, and 15 (41%) of default patients.(p < 0.01) Not surprisingly, retreatment failure was more common among patients who had failed initial treatment (24%) than among relapse (3%) or default (0%) patients (p < 0.01). Default was very common among retreatment patients at 25% overall and was particularly high among patients with initial treatment default (57%) (vs. 20% among relapse patients and 24% among failure patients (p < 0.01)). Among relapse patients, the median time from the end of initial treatment to diagnosis of relapse was 7.0 years (range 9 months to 44 years).

Only 30 (10%) of 291 retreatment patients had DST testing performed with results available in the chart. Of three patients being retreated because of initial treatment failure who were tested, all had resistance to HRS, none of five being retreated because of default from initial treatment who were tested had pan-sensitive *M. tuberculosis*, and 3 of 22 (14%) patients who were being retreated because of relapse within two years after initial TB treatment had MDR-TB.

### Risk factors for relapse, failure, or default from initial TB treatment: results of the nested case-control study

Of 291 retreatment patients, 104 (36%) had started a retreatment regimen within two years of completing or stopping initial TB treatment and were, thus, eligible for the risk factor analysis. Of these, *initial *treatment charts were available for 83 (80%), and 80 were suitable for data extraction (cases, n = 80). 266 patients with initial treatment success were included as controls (controls, n = 266). (Table 1).

**Table 1:** Patient and disease characteristics of individuals receiving standard initial tuberculosis treatment, comparing patients with treatment relapse, failure, or default (cases) to patients with treatment success without relapse (controls)

Characteristic	Cases (N = 80)	Controls (N = 266)	p-value
**At treatment initiation**			

Male gender (n, %)	65 (81)	175 (66)	< 0.01

Marital status (n, %)§			0.70
Single	32 (59)	101 (53)	
Married	21(39)	85(45)	
Other (widowed, divorced)	1(2)	4(2)	

Medical comorbidities (n,%)*	13(16)	30(11)	0.23

Cavity on chest x-ray (n,%)‡	32(46)	101(43)	0.65

Age (mean, sd)	33.2(13.6)	34.5(14.3)	0.47

Weight at treatment initiation (mean, sd)	55.1 (9.5)	56.0 (9.4)	0.46

Habits (n, %)√			
Tobacco use	18 (23)	42(16)	0.16
Alcohol use	2(3)	6(2)	0.89
Illicit drug use	6(8)	4(2)	< 0.01

**During treatment**			

Sputum smear conversion to negative by 3 months (n,%)†	28(45)	220 (87)	< 0.01

Weight gain after two months of treatment (kg, sd)**	2.6 (3.8)	3.4 (3.6)	0.09

Weight gain over initial treatment period (5-6 months) (kg, sd)**	3.8 (5.8)	6.6 (5.3)	< 0.01

Missed doses during intensive phase	14(18)	10(4)	< 0.01

Hospitalization	21(26)	38(14)	0.01

§N = 54 for cases, 190 for controls, as marital status was not consistently recorded in the charts

*Patients with no evidence of comorbid conditions in chart review were combined with those for whom the absence of cormorbidities was expressly noted.

‡N = 70 for cases, 237 for controls, as x-rays were not performed and recorded for all patients.

√Patients with no evidence of tobacco, alcohol, or illicit drug use in chart review were combined with those for whom the absence of tobacco, alcohol, or illicit use was expressly noted.

†N = 62 for cases (15 of 27 patients with default did not have sputum smear conversion data, 3 patients with relapse were culture-positive, smear-negative cases); N = 251 for controls.

**N = 65 for cases (12 default patients, 1 failure, and 2 relapse patients did not have 2-month weight data), 263 for controls at 2 months; n = 50 for cases, 257 for controls after 5-6 months of treatment

In a multivariable logistic regression analysis, patients undergoing initial treatment for TB were at higher risk of a composite endpoint of failure, default, or relapse within two years if they were male (OR = 2.29, 95% CI 1.10-4.77), failed to have sputum smear conversion to negative by 3 months of treatment (OR 7.14, 95% CI 4.04-13.2), or required hospitalization during treatment (OR 2.09, 95% CI 1.01-4.34). There was a trend towards increased risk of this composite endpoint among those with poor weight gain (less than 10% by two months of treatment) or missed doses during the intensive phase of treatment, but these differences did not reach statistical significance. Odds of the composite outcome were 4% lower for each 1 kilogram increase in weight at treatment initiation (OR 0.96, 95% CI 0.93-0.99). Alcohol use and HIV were uncommon (2% and <1%, respectively).

Risk factors appeared to differ by subgroup, though analyses were limited by sample size (Table 2). Risk factors for treatment default included male sex, substance use, missed doses during the intensive phase, and hospitalization. Risk factors for failure or relapse were harder to identify.

**Table 2:** Risk factors for relapse, failure, or default from initial TB treatment, a subgroup analysis.

Subgroup Risk factor	Univariate analysis (OR, 95% CI)	Multivariate analysis (OR, 95% CI)
**Relapse (N = 35)**		

Lack of sputum smear conversion to negative by 3 months	4.58 (2.22-9.43)	4.14 (1.92-9.01)

Missed doses during the intensive phase	3.23 (0.96-10.9)	NS

**Treatment failure (N = 18)**		

Lack of sputum smear conversion to negative by 3 months	**	**

Comorbid conditions	3.29 (1.08-9.99)	NS

**Treatment default (N = 27)**		

Male gender	6.50 (1.51-28.1)	4.56 (0.96-20.7)

Substance use†	4.19 (1.83-9.56)	2.73 (1.04-7.15)

Missed doses during the intensive phase	12.9 (4.65-35.7)	10.9 (3.47-34.0)

Hospitalization during treatment	2.56 (1.04-6.23)	3.84 (1.41-10.5)

** Three-month sputum smear conversion to negative was negative in all failure patients so could not be included in logistic regression models.

†Tobacco, alcohol, or illicit drug use.

## Discussion

In this study of 291 patients undergoing retreatment for TB, outcomes differed considerably by group -- 74% of patients with relapse, 48% of patients with failure, and 41% of patients with default had treatment success -- similar to previous studies[5,7]. Default from retreatment was extremely common at 25%, higher than previous country-wide estimates[5]. This may reflect temporal changes in treatment completion but more likely represents differences in study populations, as we focused on TB "hot spots", or urban centers with comparatively high TB incidence. Recent studies have demonstrated that, in urban settings, adherence is linked to patient knowledge about TB and provision of disease-specific education by the health care provider to the patient[8]. In busy urban clinics, time for education may be limited. Default from retreatment was most frequent among those who had defaulted from initial treatment, while failure was most common among those with previous failure. Although retreatment guidelines are often the same for patients with failure, default from, or relapse after initial treatment,[4] these results suggest that groups may benefit from different management strategies[9,10]. For example, treatment failure is commonly due to drug resistance, while recurrence may be due to poor adherence, high mycobacterial burden (such as in cavitary disease), or exogenous reinfection. Default patients may require intensified case management and education, rather than more intensive treatment.

The present study shows that, even when available, drug susceptibility testing is underutilized. It was performed in only 10% of retreatment patients. All 3 failure patients who underwent DST testing had MDR-TB, while 3 of 22 of relapse patients and 0 of 5 default patients tested did. While these DST results were only available for three failure patients and, therefore, not representative, these data and those from other studies suggest that MDR risk is not uniform among retreatment subgroups, with increased prevalence of MDR among patients with initial treatment failure[2,11-13]. According to a population-based study conducted among retreatment cases in Morocco, 12.2% had MDR-TB, but the study did not divide retreatment patients into failure, relapse, or default subgroups[6]. Taken together, these findings support use of DST in all retreatment patients, earlier DST testing in those with clinical and microbiological indications of impending treatment failure, and use of second-line drugs for retreatment of patients with initial treatment failure until DST results are known. In Morocco, DOTS coverage is 100%, and concerted efforts to dramatically enhance DST use are underway.

Published medical risk factors for failure or relapse include HIV infection, diabetes mellitus, low body weight, cavitation on chest x-ray, high bacterial burden, short treatment duration, drug resistance, and positive culture after two months of treatment[14-16]. Sociodemographic factors include unemployment, drug abuse, alcoholism, smoking, and poor treatment adherence. Treatment default is known to be associated with substance abuse, foreign birth, male gender, previous default, low socioeconomic status, psychiatric illness, unemployment, migration, side effects, [17,18] long distance to the clinic, social stigma, and poorly-implemented DOTS but, of course, differ by setting [16,19-21]. In our study population, HIV infection is rare; among TB patients, less than 1% are HIV-infected (unpublished data, Morocco NTP). Further, alcohol use in Morocco is uncommon, and smoking is extremely uncommon among women. Moreover, in the urban clinics studied, the majority of patients are non-immigrants, the clinics are geographically accessible, and DOTS coverage is 100%. Thus, many traditional risk factors for poor TB treatment outcomes are less prominent in Morocco, making it harder to prospectively identify patients at risk. However, continued sputum smear positivity after 3 months of treatment is a strong predictor of subsequent poor outcomes, and should prompt DST testing in all patients. As missed treatment doses may herald impending default, enhanced communication between the local clinics that dispense TB treatment and physicians at the regional health centers that prescribe it may be one country-specific strategy to help pinpoint those individuals who are missing doses and are at high risk of defaulting altogether. Small sample sizes limited our ability to evaluate subgroups, but even so, we were able to identify male sex, substance use (tobacco, alcohol, or illicit drug use), and missed doses during the intensive phase as likely risk factors for treatment default. Higher odds of hospitalization probably reflected the need for hospitalization to ensure adherence rather than increased disease severity. Further exploring risk factors for treatment default may help control programs identify those likely to benefit from targeted interventions such as health education, substance abuse counseling, enhanced tracking, or reinforcement of DOTS supervision[22,23].

As a retrospective chart review, our risk factor evaluation was limited by the availability of data present in clinical charts. Information about tobacco, alcohol, and illicit drug use was not routinely recorded, for example. Classifying those with missing substance use data as nonusers likely biased analyses of this risk factor toward the null; however, estimates of smoking, tobacco, and illicit drug use were similar to national substance use statistics[24]. Also, in cases of recurrence, it was not possible to distinguish between relapse and reinfection, so we limited our risk factor analysis to those who had received initial TB treatment within two years of recurrence and were, thus, more likely to have true relapse. Our ability to identify independent risk factors in subgroup analyses was limited by small sample sizes; questions regarding risk factors in these subgroups would best be answered in larger, prospective studies. Finally, DST testing was not universally performed in retreatment patients, so selection bias is possible, as clinicians are more likely to send those at high risk of resistance for testing. In our study, 20% of retreatment patients with DST had MDR-TB, compared with 12% in a national prevalence survey[6].

## Conclusions

Patients presenting for TB retreatment - those with relapse, failure of initial treatment, or default -- are often grouped together and treated with a standard Category II retreatment regimen. However, these groups have distinct demographic and clinical characteristics, important differences in retreatment outcomes, and likely have different rates of drug resistant *M. tuberculosis*. Default from retreatment is common in high-incidence urban centers in Morocco, pointing to the need for strategies to address adherence. DST is essential for identifying retreatment patients with drug resistance, but even when available, it is underutilized, likely due to practical constraints. Preventing the need for retreatment in the first place is the best strategy given the individual and public health consequences of poor initial TB treatment outcomes, so strategies to identify and address country-specific risk factors are warranted to maximize treatment success.

## Competing interests

The authors declare that they have no competing interests.

## Authors' contributions

KED designed the study and helped with data analysis, interpretation of results, and drafting of the manuscript. OL assisted with access to and interpretation of laboratory drug susceptibility testing results. IG conceived of the study and assisted with study design, interpretation of results, and editing of the manuscript and provided invaluable clinical insight. JK helped with data collection and study design and implementation. MDE assisted with access to laboratory drug susceptibility testing results. IC performed the data analysis and assisted with interpretation of results. REA assisted with study design and study implementation and provided oversight of key study personnel at INH. All authors read and approved the final manuscript.

## Pre-publication history

The pre-publication history for this paper can be accessed here:

http://www.biomedcentral.com/1471-2458/11/140/prepub
